# Calciphylaxis: A Deceiving Cellulitis

**DOI:** 10.7759/cureus.6518

**Published:** 2019-12-30

**Authors:** Sreenath Meegada, Madhavi Annakula, Tejo Challa, Prashanth Peddi

**Affiliations:** 1 Internal Medicine, The University of Texas Health Science Center/Christus Good Shepherd Medical Center, Longview, USA

**Keywords:** calciphylaxis, cellulitis, end stage renal disease

## Abstract

Calciphylaxis is a rare and serious disorder seen most in end-stage renal disease (ESRD) patients on dialysis. It is associated with the calcium deposits in small and medium blood vessels of the skin and subcutaneous tissues resulting in painful skin lesions, plaques, ulcerations, gangrene, and secondary infections. The aim of our present report is to create awareness and encourage providers to consider calciphylaxis in the differential diagnosis of cellulitis in the appropriate clinical setting.

## Introduction

Cellulitis is defined as inflammation of the skin and subcutaneous tissue. Infection is the most common cause of cellulitis; however, non-infectious causes of cellulitis should be considered when cellulitis fails to respond to conventional antibiotic treatment [1]. Failure to establish a correct diagnosis may result in unnecessary use of costly antibiotics, antibiotic toxicity, development of antibiotic-resistant bacteria, and delayed treatment of the patient’s true condition. Calciphylaxis is a potential cause of inflammation of the skin and subcutaneous tissue [2].

Calciphylaxis is a condition associated with the deposition of calcium in the tunica media of the small, medium-sized vessels of the skin and subcutis [3]. Painful erythematous skin lesions are the most common manifestation of calciphylaxis [2]. It affects 1% to 4% of people undergoing long-term hemodialysis and is associated with high morbidity and mortality [4].

## Case presentation

**Figure 1:**
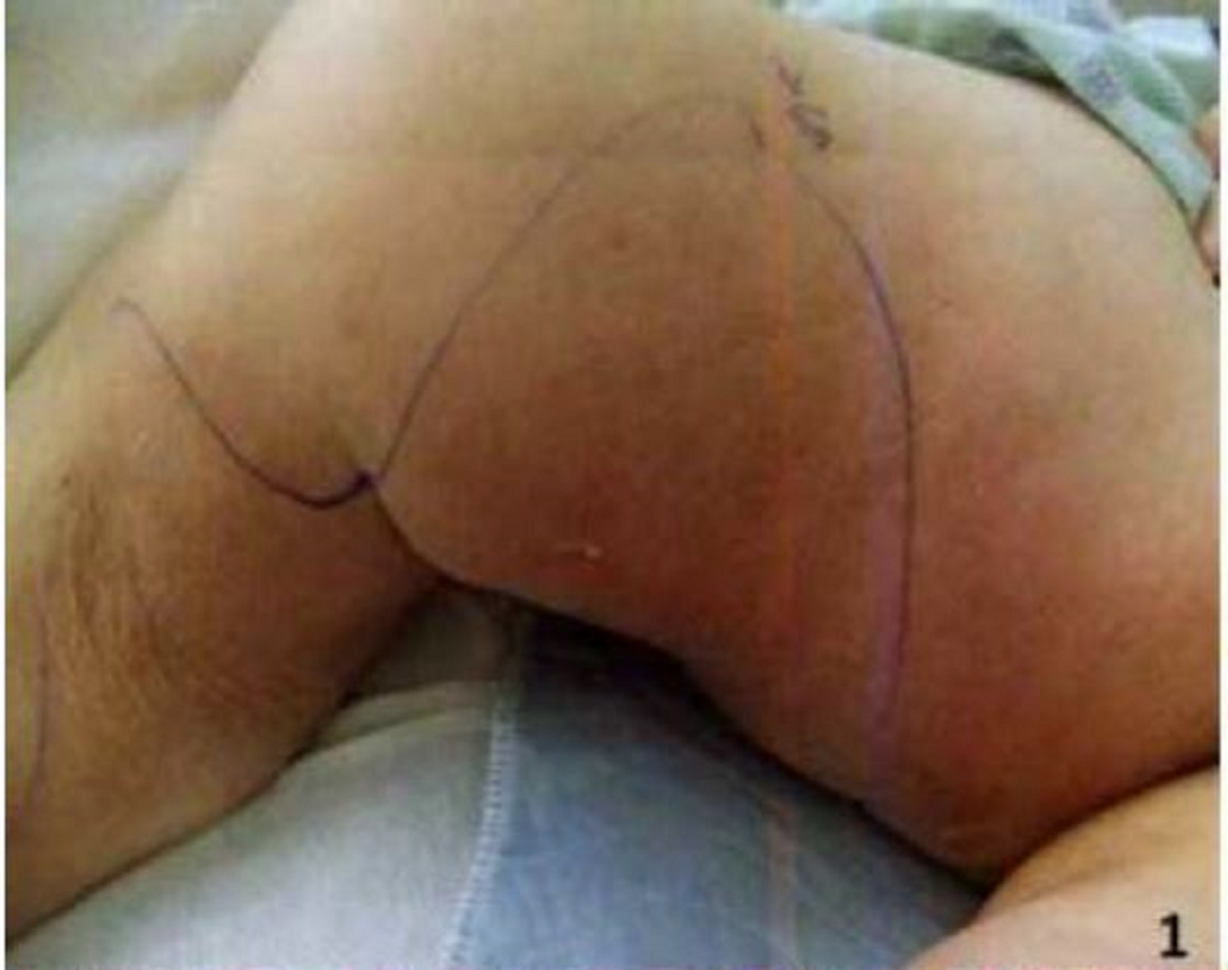
Calciphylaxis skin lesion on the right inner thigh (demarcated by markings)

**Figure 2:**
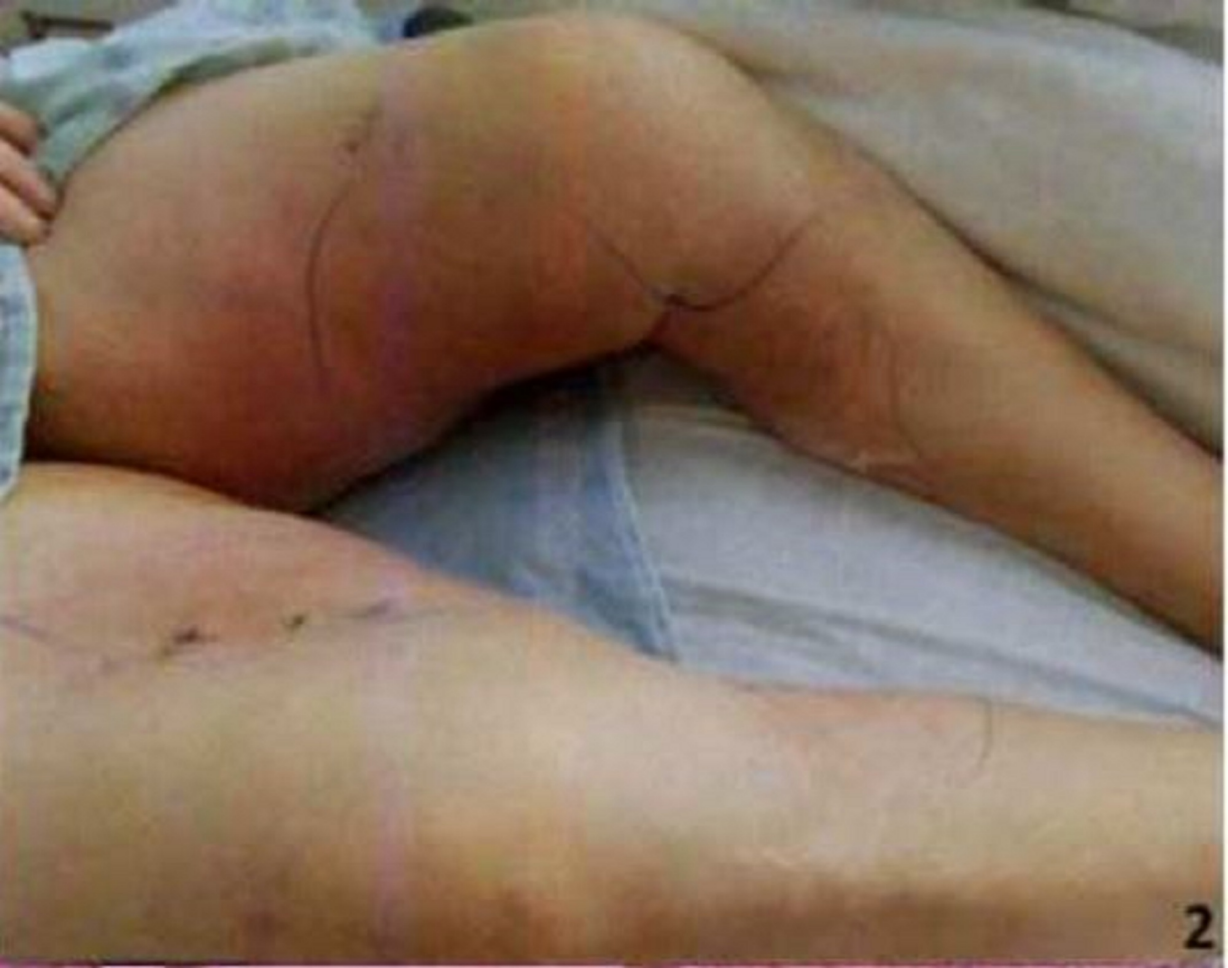
Calciphylaxis skin lesion on the left inner thigh extending to the leg (demarcated by markings)

A 53-year-old Hispanic male with poorly controlled diabetes, hemodialysis for two years, and coronary artery bypass grafting 10 months previously, developed redness pain and swelling of the medial aspect of both legs two days prior to admission. This occurred in a symmetrical fashion and extended from the upper one-third of the calf to the mid-thigh. The patient had difficulty walking secondary to pain. He had undergone saphenous vein harvesting from these areas for his coronary artery bypass graft (CABG). The patient denied trauma to the lower extremities, fevers, chills, and rigors. His medications include paricalcitol, sevelamer, calcium acetate, aspirin, amiodarone, amlodepine, simvastatin, and humilin 70/30. Physical examination showed extensive erythema involving the inner aspect of the thighs down to the medial upper one-third of the calf on both sides (Figures 1-2). Skin lesions were warm and very tender to touch, but there was no induration. Initial laboratory examination revealed white count 9,500 with 70% neutrophils, erythrocyte sedimentation rate of 87 mm/hr, C-reactive protein 11.6 mg/dl, blood urea nitrogen (BUN) 57 mg/dl, creatinine 11.3 mg/dl, calcium 9.8 mg/dl, albumin 3 gm/dl, prothrombin time (PT) 11.3 sec, international normalized ratio (INR) 1.1, and activated partial thromboplastin time (aPTT) 30.3 sec.

**Figure 3:**
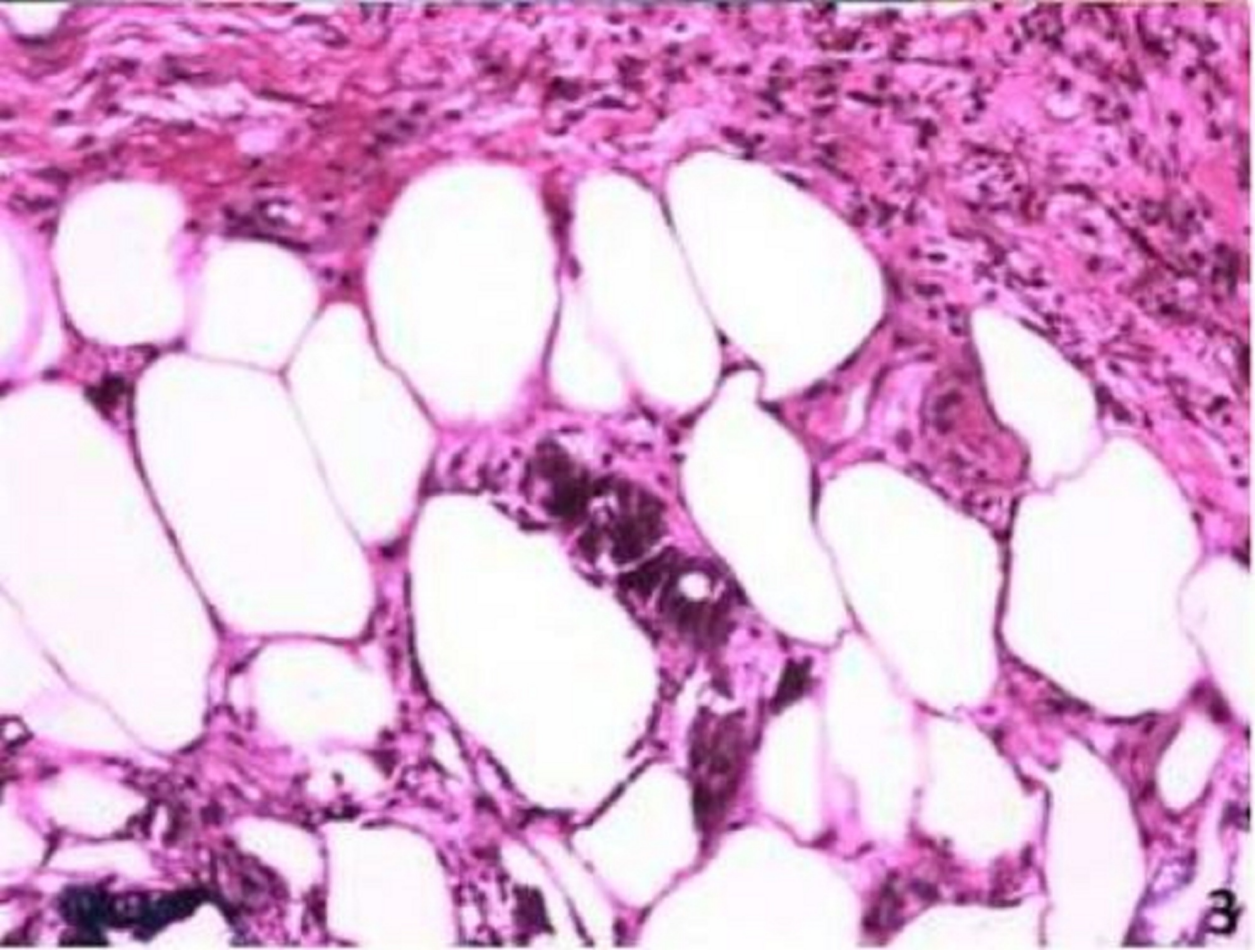
Hematoxylin and eosin (H&E) staining of skin biopsy showing calcium deposits in the small blood vessels and areas of fat necrosis with foamy histiocytes and neutrophils

**Figure 4:**
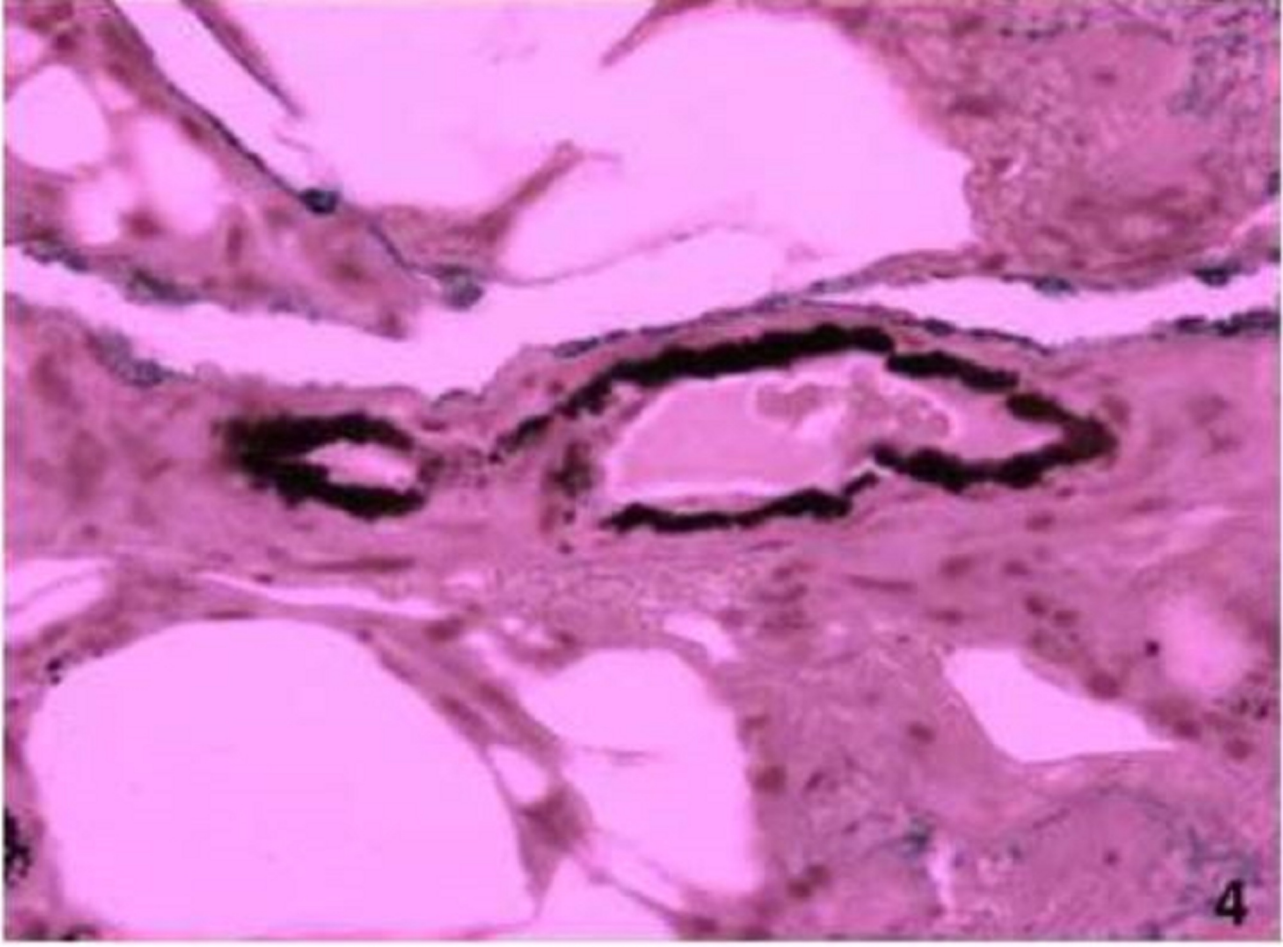
Hematoxylin and eosin (H&E) stain of skin biopsy showing clear fat necrosis, foamy histiocytes, and neutrophils

He was treated with intravenous vancomycin and ceftriaxone. By day three, moderate induration had developed, there were no signs of improvement, and the intensity of pain worsened. CT scan of both lower extremities was not consistent with necrotizing fasciitis and repeat labs showed a white count 15,700, with 75% neutrophils, and C-reactive protein 48 mg/dl, calcium 9.76 mg/dl, phosphorus 12.5 mg/dl (first time obtained). He required a patient-controlled analgesia (PCA) pump to control his pain with little improvement. His antibiotics were changed to linezolid, tobramycin, and levofloxacin. On the fourth day, there was an increased induration of the thighs with rock hard consistency of the tissue. Skin biopsy was performed showing deposition of calcium in the small blood vessels of the skin, areas of fat necrosis with foamy histiocytes, and neutrophils consistent with calciphylaxis (Figures 3-4). Parathyroid hormone level (PTH) was 628 pg/ml.

He received daily dialysis with intravenous sodium thiosulfate given post dialysis for three days. His calcium-containing phosphate binders and paricalcitol were stopped; dosage was increased for sevelamer and lanthanum carbonate, another phosphate binder, was added to lower phosphorus levels. The patient’s pain, redness, swelling and induration improved over the next three days and his calcium was 8.5 gm/dl and phosphorus 7.3 mg/dl at the time of discharge.

## Discussion

Bacterial infection is the most common cause of cellulitis in clinical practice [5]; however, there are times physicians will encounter cellulitis that does not respond to antimicrobial therapy. In addition to considering noncompliance, drug resistance and necrotizing fasciitis physicians should also consider non-infective causes of cellulitis. Our case-report is a classic example of non-infectious cellulitis. Other conditions like sarcoidosis, contact dermatitis, vasculitis, cutaneous lupus, pyoderma-gangrenosum, and cutaneous lymphoma can mimic cellulitis and should be considered in the differential diagnosis [6]. Symmetrical distribution in both legs, distribution along the vein and occurrence of erythematous patches at multiple sites simultaneously are few characteristics of non-infectious cellulitis [7]. Our patient with calciphylaxis manifested as a symmetrical kissing distribution of lesions in both legs.

The term calciphylaxis, also known as calcific uremic arteriopathy was coined by Hans Selye and based on rat experiments [8]. It is almost always seen in end-stage renal disease (ESRD) with a prevalence of 1%-4% and has a female predominance. Cases of calciphylaxis have been described in non-uremic settings, such as primary hyperparathyroidism, inflammatory bowel disease, systemic lupus erythematosus, alcoholic cirrhosis, patients with breast cancer receiving chemotherapy, and rheumatoid arthritis [9]. Risk factors identified in these cases include female sex, diabetes, obesity, calcium x phosphorus product >70, rapid weight loss and hypoalbuminemia [10].

Pathogenesis of calciphylaxis is uncertain but according to Selye experiments in rats, it was proposed that the tissues are first sensitized by PTH, vitamin-D, calcium and phosphate, after a critical period exposure to agents like local trauma, steroids, immune-suppressants, protein-c deficiency, iron-salts, and egg-albumin results in inflammation, necrosis, and tissue calcification.

The most common clinical presentation is painful, erythematous skin lesions that progress to nodular or plaque-like lesions as seen in our patient. These lesions further progress to necrotic non-healing ulcers and may develop secondary infection [11]. Lower extremity is frequently the site of involvement and proximal involvement is more common than distal. Other reported sites include the breast and penis [12]. Unusual manifestations include acral gangrene, mesenteric ischemia, and painful myopathy [13]. In patients with acral gangrene, the presence of intact peripheral pulses distinguishes it from atherosclerotic occlusive disease. Hyperparathyroidism is seen in 82% of patients with calciphylaxis [2]. Diagnosis is based on tissue biopsy, which remains the gold standard, and shows the calcium deposition in the tunica media of small blood vessels associated with intimal proliferation, fibrosis, thrombosis and fat necrosis [14]. Prognosis of calciphylaxis is poor associated with one-year mortality about 50% -60% both in uremic and non-uremic settings mainly from secondary infection and sepsis [2,9].

Our patient had multiple risk factors for calciphylaxis including diabetes, obesity, ESRD, and high calcium x phosphorus product >70. He experienced rapid onset of erythema and pain of the legs in a symmetrical distribution with progressive pain out of proportion to his clinical examination that required a PCA pump.

Treatment options proposed for calciphylaxis include attempted normalization of calcium phosphorus product to less than 55 through dialysis and removal of external calcium and phosphorus-containing compounds, intravenous sodium thiosulfate, removal of challenging/sensitizing agents, stopping warfarin (warfarin blocks matrix-glutamate carboxylase-protein, an inhibitor of vascular calcification [15]), potential parathyroidectomy, cinacalcet, and hyperbaric oxygen [16]. Our patient was treated with increased frequency of dialysis and intravenous sodium thiosulfate, stopping calcium-containing phosphate binders, maximizing the doses of sevelamer, and lanthanum carbonate. His symptoms improved markedly over three days and his skin lesions resolved when seen two months after discharge.

## Conclusions

Calciphylaxis should be considered in patients presenting with cellulitis in the setting of ESRD and high calcium x phosphorus product. Early diagnosis and intervention may prevent catastrophic complications like ulcerations, gangrene, and secondary infections.
